# Supplementary material for: STRAINS: A big data method for classifying cellular response to stimuli at the tissue scale
Source: PLoS One. 2022 Dec 8;17(12):e0278626. doi: 10.1371/journal.pone.0278626 (PMC9731430; doi:10.1371/journal.pone.0278626)
Supplement: Attachment — Submitted filename: Response_Letter_to_PLOS_One.pdf [file pone.0278626.s003.pdf]

Response to Reviewers

October 29, 2022

We thank the referees for their careful reviewing of our manuscript and providing us with valuable feedback. We are happy that reviewer 1 believed "the approach seems reasonable and the execution sound" and that the method falls in line with our goals, where the "code provided is fully documented and incorporates a GUI for non-programmers". Reviewer 1 asked that we demonstrate that the tool can be used on other systems, which we have addressed by applying the technique to a new dataset, tracking and classifying the neural responses of worms [1]. While Reviewer 2 felt that "Without an unambiguous meaning and interpretation on the basis of additional solid data, a classification of cell responses using descriptive signatures as provided by the STRAINS software has little scientific value," we would like to emphasize that the purpose of this manuscript is to outline and demonstrate the potential of the STRAINS method. The purpose of the STRAINS tool is to enable future experiments where identification of categories that represent specific behaviors can be used to further our understanding of cellular responses. Here, however, we do not attempt to establish any biological insights within this manuscript, since it is focused on the tool itself, rather than applications of the tool for scientific discovery. The goal of this submission is to give other researchers the opportunity to use and build upon the STRAINS method, similar to the numerous manuscripts published in PLOS ONE demonstrating tools and methods that could be used for future scientific discovery [2–17].

In addition, we respond to each specific comment raised by the editor and reviewers:

1 Response to Editor's Comments

1. Please ensure that your manuscript meets PLOS ONE's style requirements, including those for file naming. The PLOS ONE style templates can be found at https://journals.plos.org/plosone/s/file?id=wjVg/PLOSOne_formatting_sample_main_body.pdf and https://journals.plos.org/plosone/s/file?id=ba62/PLOSOne_formatting_sample_title_authors_affiliations.pdf

We have used the PLOS ONE latex template as found on the website https://journals.plos.org/plosone/s/latex.

With this response, we have included both the initial manuscript and the modified manuscript with new additions to the text in blue and removed text struck out in red. We note that these changes were tracked for changes to the text, but additionally, due to the insertion of an additional citation, citations after 37 have been shifted by one number. Changes to the citation numbers were not highlighted in red/blue to avoid confusion.

2. We note that the grant information you provided in the 'Funding Information' and 'Financial Disclosure' sections do not match.

We have removed the Acknowledgements section and updated the funding information to correctly match the financial disclosure section. The correct statement is as follows:

"Funding: The work was supported by the NIH National Institute of Arthritis and Musculoskeletal and Skin

Diseases (niams.nih.gov), Contract: 5R01AR071394 to LAF, K08AR068470 to MLD, R03AR075929 to MLD, and The Harry M. Zweig Fund for Equine Research to LAF and MLD. Additionally, this work was supported by the National Science Foundation (nsf.gov) grants DMR-1807602 to IC and LJB, CMMI-1927197 to LJB and IC, and BMMB-1536463 IC and LJB. Lastly, this work made use of the Cornell Center for Materials Research Shared Facilities which are supported through the NSF MRSEC program (DMR-1719875). The funders had no role in study design, data collection and analysis, decision to publish, or preparation of the manuscript."

2 Response to Reviewer 1

1. I want to address that the paper claims that the software can be used for a wide variaty of data. However, the autors show only one measuerement one bovine certilage of 5 ROIs and the GUI is customized to this measurement type. Given the claim to be multi-purpose, exspecially lines 301 to 312, I would have appreciated to see the application to 1-2 other, probably openly available, datasets in the supplementary material.

Response: We agree that additional analyses using our system would further cement the effectiveness of the STRAINS technique. We have added a section to the supplementary information detailing the use of STRAINS on GCaMP and neural activity in freely moving Caenorhabditis elegans roundworms from Wen, et. al. eLife, 2021 [1]. We updated the GUI code to enable automatic detection of image size in order to accommodate the shape of the video, and used it to analyze single channel data from that Wen, et. al. Figs. 1 and 2 show the updated GUI video panel alongside single and multiple cell plots on the right panel.

Additionally, we applied our unmodified time series classification technique to neural activity data supplied by the authors, reaching classification accuracies up to 91.2% for all four classifiers (CIF, DrCIF, ROCKET, Arsenal), where the maximum accuracy is the same for all categories due to the smaller sample size (137 cells). We note that based on this analysis, heatmaps similar to those seen in Fig. 4 (in the manuscript) were generated, which would allow for the potential generation of hypotheses based on mapped neuronal behaviors, but the authors have requested that we do not make this analysis public as they have not yet published their biological conclusions based on this data.

Supplementary Information, Example Application of STRAINS to Worm Neuron Data, Page 4: We tested our system on data obtained from Wen, et. al. *eLife*, 2021 [1]. Using GCaMP and calcium dynamics data from Fig. 5 in that publication, we applied our modified particle tracking to the video of a moving worm brain. Our GUI was modified to automatically accommodate the new video dimensions, tracking, and single channel data (Fig. 1 and Fig. 2). Based on manual classification, unmodified time series classification was trained and tested on calcium dynamics data, reaching classification accuracy of up to 91.2% for all four classifiers, CIF, DrCIF, Arsenal, and ROCKET (similar accuracy is obtained for all classifiers due to small population size of data - 137 cells total).

2. The peak stress of "approximately 1 MPa" is mentioned and a "wide range of strains" accross the sample. As the stress and strain exerted seems crucial for the biological evaluation of such data, a proposal of strain/stress measurements or a proposal for theoretical strain/stress modelling for this data would improve on the method.

We understand that measurement of the strain field as brought up by Reviewer 1 was not addressed in the manuscript. We have previously reported on the strain measurements associated with this apparatus in Bartell et. al., 2015 and Henak et. al., 2017. We have updated the methodology to include these measurements.

Subsection Experimental system and *in situ* imaging procedure, Paragraph 1, Page 5: One sample was used as a control while the second sample was impacted to induce injury. The impactor was calibrated to deliver to the articular surface a 5-10 ms impact with a peak stress of $\sim$1 MPa, which produced superphysiologic strains and strain rates encompassing the wide range of strains observed in joints with traumatic injury within a small field of view [18–20]. Strain calibration was conducted with a high speed

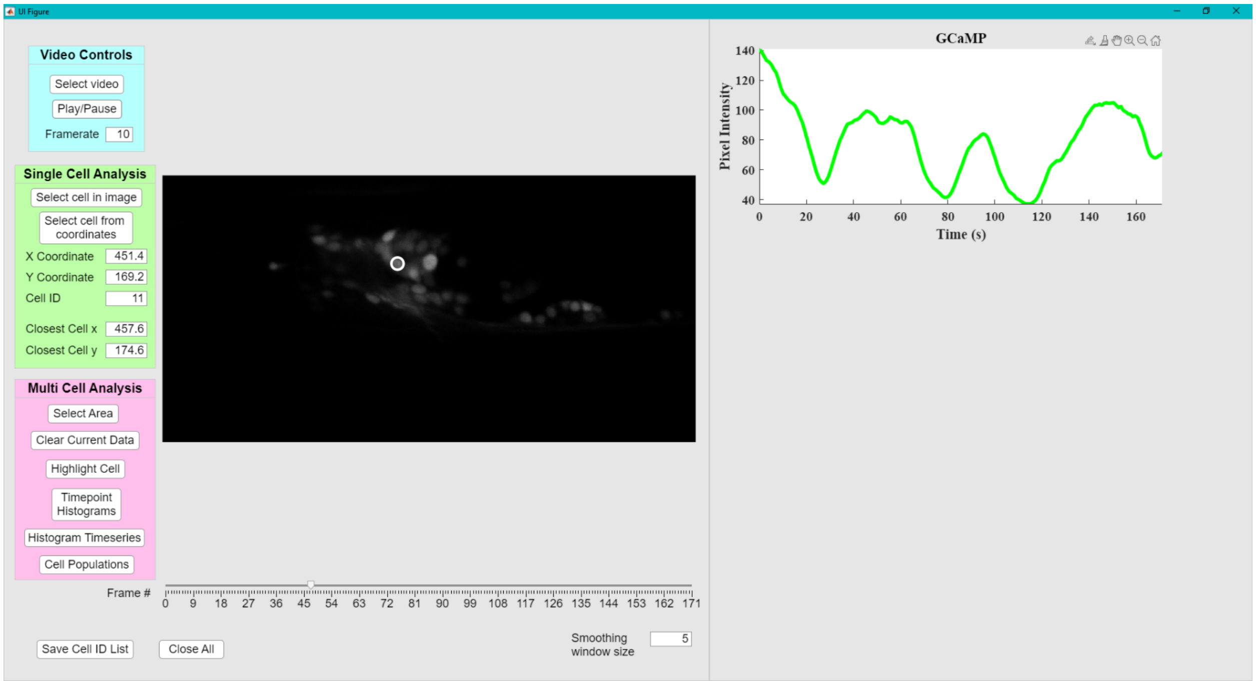

Figure 1: Example screen capture of modified GUI on worm GCaMP data for selecting single cells within the video.

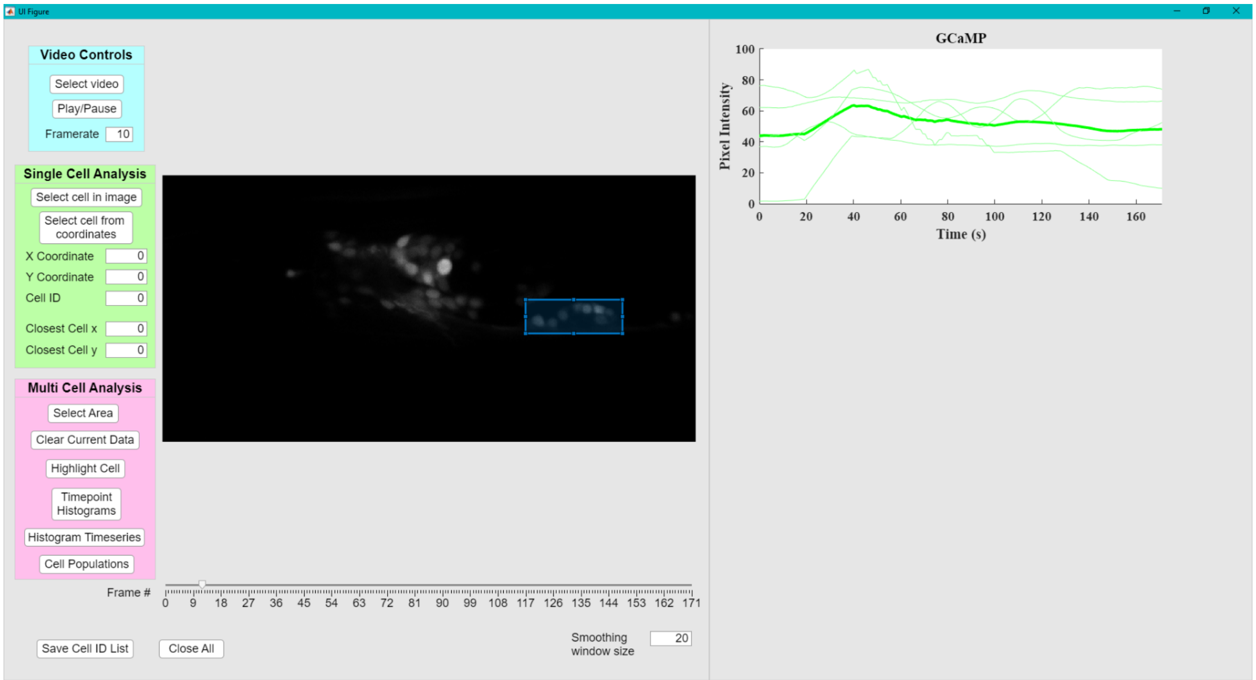

Figure 2: Example screen capture of modified GUI on worm GCaMP data for selecting groups of cells within the video.

camera (v7.1, Vision Research), as described in Bartell et. al., 2015 and Henak et. al., 2017. To create visual texture for measuring strain fields, the cut surface of the impacted sample was coated with fluorescent polystyrene microspheres (2 μm carboxylate particles). During impact, cartilage deformation was recorded using the high speed camera at 1000 frames per second. Digital image correlation was used to track the deformation and calculate strain fields.

3. During the paper, the description of the experimental setup and locations in the tissue seemed unprecise and confusing. Examples are "outward from the injury site", "to the side/below the impact side"... "cells below the injury site". Mentioning relation to the surface level of the sample or the depth of the tissue would be helpful.

Subsection Experimental system and *in situ* imaging procedure, Paragraph 2, Page 5: This entire apparatus was loaded onto a fast confocal microscope that enabled imaging of the cells throughout the impact and relaxation process. Each region corresponding to the field of view for our 10X objective was 660 μm x 660 μm in size. We assessed multiple regions to understand the influence of a wide range of local tissue strains on the behaviors of cells (Fig. 1d). On the impacted sample, we imaged ~~directly at~~ the entirety of the impact site (region 1) at the surface of the tissue from 60 μm above the articular surface to 600 μm below the surface (superficial zone of articular cartilage), ~~to the side of~~ lateral to and at the same depth as the impact site (region 2), also at the surface of the tissue, and directly below the impact site (region 3), in the region extending 600 μm to 1260 μm below the surface (middle zone of articular cartilage). On the unimpacted control, we imaged two sites (regions 4 and 5) at the same tissue depths (superficial and middle zones) as the impacted sample in order to compare cells of similar phenotype. From this imaging process, we obtained the fluorescent intensities of each cell (expanded image in Fig. 1d).

Fig. 2 Caption, Page 6: Images are shown at the impact site (region 1), with the impact location labeled with an orange arrow. a) High speed imaging of calcium concentration in the minute after impact showed a wave of calcium uptake in cells moving outward from the immediate impact site. Inset shows the area experiencing mostly shear strain, with a more muted and delayed response in comparison with areas experiencing compressive strain. b) Long term imaging of all three stains (mitochondrial polarization, calcium concentration, nuclear membrane permeability). As time progressed, mitochondrial polarization and calcium concentration slowly decreased while nuclear membrane permeability progressively increased with greatest intensity ~~at the impact site~~ within 400 μm of the impact site.

Subsection Strain dependent cellular response, Paragraph 1, Page 6: The strain field resulting from impact and the associated cell response had complex behaviors that varied spatiotemporally. Specifically, we observed complex patterns in the immediate post-impact Ca^{2+} response and hours-long cellular responses for all three measured signals. In the milliseconds after impact trauma, increased calcium concentration can be observed in cells proximal to the impact site, in the superficial region. However, on the seconds timescale, we observed differences in total intensity between chondrocytes experiencing shear and compression within this region (Green in Fig. 2a). On longer time scales, we found that mitochondrial polarity rapidly diminished at the impact site in the minutes after injury, with calcium concentration following the same pattern but with some cells exhibiting transients on the scale of minutes (Supplementary Video). Conversely, nuclear membrane permeability initially showed a very low intensity throughout the region and reached higher intensities in a fraction of the cells in regions extending up to 400 μm below the impact site on a time scale of hours. Consistent with the short time calcium response, this pattern of cell death did not extend to areas of the tissue which experienced primarily shear strains.

3 Response to Reviewer 2

These maps show for example that the impact site has the highest number of cells with transient increase in membrane permeability. The row of cells behind the impact site show a calcium transient with subsequent increase in nuclear permeability, and the cells in the south-east corner diagonal behind the impact site show a calcium transient but no change in nuclear permeability.

We are pleased that reviewer 2 was able to easily identify and interpret the classifications presented by our method.

The development of the software was motivated by the belief of the authors that "the sheer scale" of collected data was not amenable to typical statistical analyses. Where this belief comes from, what is meant by "typical statistical analysis", or the scale of data beyond which the authors think typical statistical analysis might fail, remains unexplained.

While there are many standard tools for imaging cellular behavior, many are for single snapshots and not amenable to tracking thousands of cells through deforming tissues. For example, ImageJ has plugins that allow for manual tracking of moving objects or automated segmentation of cells and individual cell tracking (TrackMate), but does not incorporate tracking associated with tissue deformation or the extraction of multi-channel fluorescent time traces. Further, these plugins are not interactable in a way similar to our GUI, which allows the parsing of thousands of cells within a single frame simultaneously.

We are also specifically interested in how signals change over time, instead of broad population statistics like mean and standard deviation. Existing techniques are unable to pick out specific time series characteristics (such as peaks, peak shape, changepoints, etc), or interactions between multiple different fluorescent channels tracking different components of cellular function.

Additionally, there are no techniques utilizing these state-of-the-art multivariate time series classification algorithms (sktime) that have only recently been developed, in application to time series of cellular functions. For large numbers of cells, automated classifications schemes are necessary.

To make the limitations in currently available tools more explicit, we have updated the text:

Subsection Enhanced particle tracking captures behaviors of moving cells, Paragraph 1, Page 7: Importantly, the sheer scale of data made it prohibitively difficult to use typical statistical analyses or cell tracking tools, which are limited in tracking capabilities and unable to pick out specific time series characteristics and interactions between multiple different fluorescent channels tracking different components of cellular function. In order to interpret our data, ~~however~~, we needed to categorize cellular signatures, relate each cells' response to its location within the tissue, and determine whether the cellular signals from multiple cells were spatiotemporally clustered.

Some of these observations appear trivial, others are puzzling, but none of these observations contribute to any new insight, or to an understanding of how cells collectively coordinate their response to a mechanical stimulus.

We agree with Reviewer 2 that some of these "observations appear trivial" and "others are puzzling", as any analysis of all cells within a region of tissue will result in a subset of observed cells displaying trivially understood behaviors. We believe that the ability of STRAINS to quickly identify and sort between trivial and puzzling behaviors is what allows us to quickly form hypotheses and test these puzzling behaviors in future experiments. In order to fully analyze and categorize all cells, observations of these trivial behaviors must be made, and further, allow us to focus more effectively on puzzling behaviors which we do not understand.

Subsection A MATLAB GUI for image processing, Paragraph 4, Page 9: Importantly, the precision with which we measure the cell intensities and correlations in cell signatures allowed for distinguishing between these behaviors, even when the accompanying curves were quite similar. For example, in some cells the calcium transients were not associated with an increase in nuclear membrane permeability (Fig. 4i) while in others, the calcium transient occurred immediately before a rise in nuclear membrane permeability (Fig. 4h) or at some time after the nuclear membrane permeability rose (Fig. 4g). The differences in how these two channels interacted, along with the distinct spatial distributions associated with each behavior, indicated that separate biochemical processes associated with activation of calcium channels may have taken

place illustrating the power of our approach for generating a comprehensive map of the tissue scale multi-channel cellular response to an applied perturbation. By quickly identifying and spatiotemporally mapping behaviors ranging from normal to abnormal, our approach allows us to focus on interesting and puzzling behaviors for future experimentation.

Also, the speculation which categories represent fingerprints of Piezo activation versus TRPV4 activation is far-fetched and not helpful, as this was also not measured. Without an unambiguous meaning and interpretation on the basis of additional solid data, a classification of cell responses using descriptive signatures as provided by the STRAINS software has little scientific value.

This manuscript is intended as a showcase of the technique and claims no scientific results. We understand what Reviewer 2 is suggesting, that these specific hypotheses do not further the goal of the manuscript. To make our intention that this is a tool to generate hypotheses rather than establishing a scientific result, we removed the paragraph pertaining to hypotheses about TRPV4 and Piezo 1/2.

Discussion, Paragraph 2, Pages 13-14: ~~A powerful application of this technique is the sharpening of novel observations into hypotheses related to specific signaling pathways. For example, previous work has suggested that the mechanosensitive ion channels Piezo 1/2 are activated by *superphysiologic* loading and induce catabolic cellular responses leading to tissue degradation and pathologies like osteoarthritis. Using the STRAINS tool, we identified cells below the impact site which experienced compression and demonstrated calcium transient(s) in the milliseconds after impact followed by increased nuclear membrane permeability in the subsequent hours (Fig. 4h), a behavior likely dominated by Piezo 1/2 signaling. STRAINS would allow us to probe this hypothesis in future experiments, for examples by using Piezo 1/2 agonists and antagonists to determine how these interventions change cellular behavior patterns. Relating how a given intervention mitigates or exacerbates existing behavior categories, or establishes new behaviors would thus enable us to test mechanistic hypotheses related to known signaling pathways, alongside observing downstream effects. In contrast, TRPV4 is an ion channel known to be activated during *physiologic* loading and to initiate beneficial anabolic cellular responses. Away from the impact site, where tissue strains were more moderate, we identified cells that experienced calcium transient(s) without an increase in nuclear membrane permeability, a behavior likely dominated by TPRV4 signaling. While TRPV4 is known to be activated by physiologic loading, how the chondrocytes coordinate their responses in space and time is not fully understood; neighboring cells can differ dramatically in their responses, despite existing in very similar microenvironments. Using STRAINS, we can quickly examine and probe these behaviors within the tissue, opening the door to understanding how cell behaviors are related to stimuli and the tissue environment.~~

Perhaps most unfortunate is that the empirical classification is not or only marginally meaningful. For example, it is unclear which categories are associated with cell death, as this was not specifically measured.

The cells subject to impact trauma largely fall into four categories: cells with minimal response to mechanical perturbation, cells that respond transiently and remain healthy, cells that show chronic dysfunction, and cells that ultimately die. Within each of these categories is a wide range of behaviors. The challenge is to understand what influences a cell to undergo a specific pathway, the first step in a more complete understanding of mechanotransduction in these systems. We note that categories can be modified at will to accommodate whatever is being tested. While these empirical observations may not drive a concrete understanding without further experimentation, they provide the foundation for establishing how cellular behaviors change in future experiments.

In addressing the specific concerns about cell death measurements, we note that nuclear membrane permeability measured with Sytox Blue has been successfully used by our group in previous experiments assessing the role of mitoprotective therapy in preventing mitochondrial depolarization and cell death [19]. Additionally, other groups studying phases of cell death have established nuclear membrane permeability as a reliable indicator of cell death [21, 22]. Further, we have conducted calibration experiments (as outlined in Methods, subsection Fluorescent intensity validation) that have demonstrated the effectiveness of nuclear membrane

permeability as a stand in for cell death. In experiments where specific definitions of cell death is strictly necessary, further calibration experiments can be conducted using standard cell death stains (i.e. Ethidium Homodimer-1), which can be seamlessly implemented with the STRAINS technique. Finally, we suggest that the STRAINS method can be customized to the needs of the user, allowing researchers to select the necessary stains corresponding to the required measurements.

To clarify the goal reasoning behind empirical observations made in the manuscript text, we updated the discussion:

Discussion, Paragraph 1, Page 13: However, by using STRAINS to continuously collect cell behavior data after impact, we were able to observe nine categories of behavior associated with increased nuclear membrane permeability, which suggested different pathways to cell death (Fig. 4a-h,l). Here, the nuclear membrane permeability increased and decreased (Fig. 4c), or increased and plateaued (Fig. 4c), indicative of cell death due to superphysiologic strain. Further from the impact site we observed multiple behaviors related to elevated nuclear membrane permeability, but with additional signals suggesting other biological mechanisms at work. For example, a subset of cells displayed multiple levels of nuclear membrane permeability (Fig. 4l), which may reflect multiple inputs or different stages of cell death processes. Collectively, such results open the door to analyzing how cellular responses are coordinated at the tissue scale. These empirical observations do not drive a concrete understanding without further experimentation, but provide the foundation for establishing how cellular behaviors change in future experiments aimed at probing specific ion channels, mitochondrial function, senescence, and other processes.

References

1. Wen, C. *et al.* 3DeeCellTracker, a deep learning-based pipeline for segmenting and tracking cells in 3D time lapse images. *eLife* **10** (eds Zimmer, M. & Calabrese, R. L.) Publisher: eLife Sciences Publications, Ltd, e59187. ISSN: 2050-084X. https://doi.org/10.7554/eLife.59187 (2022) (Mar. 2021).

2. Fan, B., You, J., Suo, Y. & Qian, C. A novel and sensitive method for determining vitamin B3 and B7 by pre-column derivatization and high-performance liquid chromatography method with fluorescence detection. en. *PLOS ONE* **13.** Publisher: Public Library of Science, e0198102. ISSN: 1932-6203. https://journals.plos.org/plosone/article?id=10.1371/journal.pone.0198102 (2022) (June 2018).

3. Zhao, H., Beck, B., Fuller, A. & Peatman, E. EasyParallel: A GUI platform for parallelization of STRUCTURE and NEWHYBRIDS analyses. en. *PLOS ONE* **15.** Publisher: Public Library of Science, e0232110. ISSN: 1932-6203. https://journals.plos.org/plosone/article?id=10.1371/journal.pone.0232110 (2022) (Apr. 2020).

4. Katano, I., Harada, K., Doi, H., Souma, R. & Minamoto, T. Environmental DNA method for estimating salamander distribution in headwater streams, and a comparison of water sampling methods. en. *PLOS ONE* **12.** Publisher: Public Library of Science, e0176541. ISSN: 1932-6203. https://journals.plos.org/plosone/article?id=10.1371/journal.pone.0176541 (2022) (May 2017).

5. Goktug, A. N., Ong, S. S. & Chen, T. GUItars: A GUI Tool for Analysis of High-Throughput RNA Interference Screening Data. en. *PLOS ONE* **7.** Publisher: Public Library of Science, e49386. ISSN: 1932-6203. https://journals.plos.org/plosone/article?id=10.1371/journal.pone.0049386 (2022) (Nov. 2012).

6. Hameed, S. S., Hassan, R., Hassan, W. H., Muhammadsharif, F. F. & Latiff, L. A. HDG-select: A novel GUI based application for gene selection and classification in high dimensional datasets. en. *PLOS ONE* **16.** Publisher: Public Library of Science, e0246039. ISSN: 1932-6203. https://journals.plos.org/plosone/article?id=10.1371/journal.pone.0246039 (2022) (Jan. 2021).

7. Cañas-Duarte, S. J., Restrepo, S. & Pedraza, J. M. Novel Protocol for Persister Cells Isolation. en. *PLOS ONE* **9.** Publisher: Public Library of Science, e88660. ISSN: 1932-6203. https://journals.plos.org/plosone/article?id=10.1371/journal.pone.0088660 (2022) (Feb. 2014).

8. Paulose, T. *et al.* SAMA: A Method for 3D Morphological Analysis. en. *PLOS ONE* **11.** Publisher: Public Library of Science, e0153022. ISSN: 1932-6203. https://journals.plos.org/plosone/article?id=10.1371/journal.pone.0153022 (2022) (Apr. 2016).
9. Langford, S. T. *et al.* Three-dimensional spatiotemporal tracking of fluorine-18 radiolabeled yeast cells via positron emission particle tracking. en. *PLOS ONE* **12.** Publisher: Public Library of Science, e0180503. ISSN: 1932-6203. https://journals.plos.org/plosone/article?id=10.1371/journal.pone.0180503 (2022) (July 2017).
10. Schall, M. *et al.* A 3D two-point method for whole-brain water content and relaxation time mapping: Comparison with gold standard methods. en. *PLOS ONE* **13.** Publisher: Public Library of Science, e0201013. ISSN: 1932-6203. https://journals.plos.org/plosone/article?id=10.1371/journal.pone.0201013 (2022) (Aug. 2018).
11. Johnstone, A. D. *et al.* A novel method for quantifying axon degeneration. en. *PLOS ONE* **13.** Publisher: Public Library of Science, e0199570. ISSN: 1932-6203. https://journals.plos.org/plosone/article?id=10.1371/journal.pone.0199570 (2022) (July 2018).
12. Doncic, A., Eser, U., Atay, O. & Skotheim, J. M. An Algorithm to Automate Yeast Segmentation and Tracking. en. *PLOS ONE* **8.** Publisher: Public Library of Science, e57970. ISSN: 1932-6203. https://journals.plos.org/plosone/article?id=10.1371/journal.pone.0057970 (2022) (Mar. 2013).
13. Soares-Bezerra, R. J. *et al.* An Improved Method for P2X7R Antagonist Screening. en. *PLOS ONE* **10.** Publisher: Public Library of Science, e0123089. ISSN: 1932-6203. https://journals.plos.org/plosone/article?id=10.1371/journal.pone.0123089 (2022) (May 2015).
14. Zhai, Y., Davenport, B., Schuetz, K. & Pappu, H. R. An on-site adaptable test for rapid and sensitive detection of Potato mop-top virus, a soil-borne virus of potato (Solanum tuberosum). en. *PLOS ONE* **17.** Publisher: Public Library of Science, e0270918. ISSN: 1932-6203. https://journals.plos.org/plosone/article?id=10.1371/journal.pone.0270918 (2022) (Aug. 2022).
15. Callenberg, K. M. *et al.* APBSmem: A Graphical Interface for Electrostatic Calculations at the Membrane. en. *PLOS ONE* **5.** Publisher: Public Library of Science, e12722. ISSN: 1932-6203. https://journals.plos.org/plosone/article?id=10.1371/journal.pone.0012722 (2022) (Sept. 2010).
16. Jo, S., Kim, T. & Im, W. Automated Builder and Database of Protein/Membrane Complexes for Molecular Dynamics Simulations. en. *PLOS ONE* **2.** Publisher: Public Library of Science, e880. ISSN: 1932-6203. https://journals.plos.org/plosone/article?id=10.1371/journal.pone.0000880 (2022) (Sept. 2007).
17. Tanaka, T. *et al.* Development of a method for measuring spleen stiffness by transient elastography using a new device and ultrasound-fusion method. en. *PLOS ONE* **16.** Publisher: Public Library of Science, e0246315. ISSN: 1932-6203. https://journals.plos.org/plosone/article?id=10.1371/journal.pone.0246315 (2022) (Feb. 2021).
18. Bartell, L. R., Xu, M. C., Bonassar, L. J. & Cohen, I. Local and global measurements show that damage initiation in articular cartilage is inhibited by the surface layer and has significant rate dependence. en. *Journal of Biomechanics* **72,** 63–70. ISSN: 00219290. https://linkinghub.elsevier.com/retrieve/pii/S0021929018301398 (2019) (Apr. 2018).
19. Bartell, L. R. *et al.* Mitoprotective therapy prevents rapid, strain-dependent mitochondrial dysfunction after articular cartilage injury. en. *Journal of Orthopaedic Research* **38,** 1257–1267. ISSN: 1554-527X. https://onlinelibrary.wiley.com/doi/abs/10.1002/jor.24567 (2020) (2020).
20. Henak, C. R., Bartell, L. R., Cohen, I. & Bonassar, L. J. Multiscale Strain as a Predictor of Impact-Induced Fissuring in Articular Cartilage. *Journal of Biomechanical Engineering* **139.** ISSN: 0148-0731. https://doi.org/10.1115/1.4034994 (2022) (Jan. 2017).
21. Truernit, E. & Haseloff, J. A simple way to identify non-viable cells within living plant tissue using confocal microscopy. *Plant Methods* **4,** 15. ISSN: 1746-4811. https://doi.org/10.1186/1746-4811-4-15 (2021) (June 2008).

22. DiPeso, L., Ji, D. X., Vance, R. E. & Price, J. V. Cell death and cell lysis are separable events during pyroptosis. en. *Cell Death Discovery* **3.** Number: 1 Publisher: Nature Publishing Group, 1–10. ISSN: 2058-7716. https://www.nature.com/articles/cddiscovery201770 (2022) (Nov. 2017).
